# Gingivitis and Its Causes in Children Aged 3–7 Years

**DOI:** 10.3390/diagnostics14232690

**Published:** 2024-11-29

**Authors:** Dorota Olczak-Kowalczyk, Anna Turska-Szybka, Marcin Studnicki, Paula Piekoszewska-Ziętek

**Affiliations:** 1 Department of Paediatric Dentistry, Medical University of Warsaw, 02-091 Warsaw, Poland; pedodoncja@wum.edu.pl (D.O.-K.); anna.turska-szybka@wum.edu.pl (A.T.-S.); 2 Department of Biometry, Warsaw University of Life Sciences, 02-776 Warsaw, Poland; marcin_studnicki@sggw.edu.pl

**Keywords:** gingivitis, children, dietary habits, hygienic habits

## Abstract

Objectives: Gingivitis manifests as redness, swelling, and bleeding of the gingiva but no loss of connective tissue attachment. It is usually painless and rarely leads to spontaneous bleeding, and most patients are unaware of the disease or are unable to recognize it. In children and adolescents, it is most often caused by plaque accumulation. The purpose of the following study was to determine the prevalence of gingivitis and its causes in children aged 3 to 7 years. Methods: Patients were classed in the following three age groups: 3-year-olds, 5-year-olds, and 7-year-olds, who were generally healthy, not taking permanent medication, and without developmental defects of dentition were eligible for the study. A questionnaire survey assessed socioeconomic factors, frequency of dental visits, and hygiene and dietary habits. The clinical examination assessed the condition of the teeth based on dmft/DMFT, and the presence of gingivitis was based on the bleeding on probing. The obtained results were subjected to statistical analysis. Results: A total of 3558 patients were examined. Gingivitis was present in 436 (12.25%) of the patients. In the group of 3-year-olds, gingivitis was significantly more common in boys (*p* = 0.0024). There were significant positive correlations between gingivitis and the average number of teeth affected by caries for the male gender in the group of 5- and 7-year-olds and in all age groups with dmft/DMFT > 0 values and the occurrence of symptomatic visits. Conclusions: The prevalence of gingivitis in children aged 3–7 years is influenced by socioeconomic, oral hygiene, and diet-related factors. Poor dental health predisposes to the occurrence of gingivitis.

## 1. Introduction

Gingivitis is characterized by redness, swelling, bleeding of gums, and no clinical attachment loss (CAL) [1]. It is usually painless and rarely leads to spontaneous bleeding, and most patients are unaware of the disease or are unable to recognize it. Results of epidemiological studies indicate that the cause of gingivitis in children and adolescents is most often plaque. Gingivitis caused by plaque is a reversible form of periodontal disease [2,3]. Poor oral hygiene and plaque accumulation are known and important predisposing factors for gingivitis. Plaque accumulation in childhood and adolescence may be associated with the risk of developing periodontal disease in later life [4]. Therefore, in the prevention of periodontal disease, a significant issue is the maintenance of proper oral hygiene in childhood. This is the time for the formation of proper hygiene habits and health-promoting behaviors. Their implementation can be helpful in preventing not only gum disease, but also caries [3,4,5].

Many epidemiological studies use the Löe Gingival Index (GI) and the Community Periodontal Index of Treatment Needs (CPITN) to assess gingival or periodontal status [6,7]. The prevalence of gingivitis was mostly defined as the presence of gingival bleeding in at least one site or sextant [7,8]. Recently, a new definition of gingivitis has been published, as a consensus of the AAP (American Academy of Periodontology) and EFP (European Federation of Periodontology) [9], which sets the threshold for gingivitis as the presence of ≥10% bleeding sites. Patients with a bleeding on probing (BOP) score of <10%, without loss of connective tissue attachment and radiographic bone loss, are considered clinically healthy. This definition is also recommended for epidemiological studies [9].

Studies on gingival status and gingival bleeding in children are few, although such knowledge can be useful in planning preventive programs [3]. Epidemiological studies conducted to date, based on the CPITN index and clinical parameters, suggest that gingivitis is a significant problem that can affect up to approx. 70% of the pediatric population [5]. In accordance with the values obtained from the CPITN, the treatment needs of the population can be estimated corresponding to the suggestions given by WHO. Therefore, healthy children (0 CPITN) do not need periodontal treatment. Children presenting with gingival hemorrhage (1 CPITN) require oral hygiene instruction. Lastly, children having gingival calculus (2 CPITN) not only require oral hygiene instruction, but also supragingival and subgingival scaling. Some researchers also point out the impact of gingivitis or periodontitis on oral health-related quality of life [8]. According to Krisdapong et al. [8], the impacts of calculus and/or gingivitis on children’s OHRQoL were mostly at low levels of extent and intensity. At a moderate/higher level of condition-specific impacts, the significant associations were with extensive calculus and/or gingivitis affecting three or more sextants in 12-year-olds, and for only extensive gingivitis in 15-year-olds.

The clinical relevance of determining gingivitis in children is significant for several reasons, as this condition can have both short-term and long-term implications for a child’s oral and overall health. Gingivitis is the initial stage of periodontal disease. Early detection in young children can prevent progression to more severe conditions, such as periodontitis, which can result in tooth loss and affect the underlying bone structure. In recent years, research has demonstrated connections between oral health and systemic conditions, even in children. For instance, untreated gingivitis can lead to increased systemic inflammation, which may influence conditions such as cardiovascular health, diabetes, or even respiratory infections [10].

The aim of the following study was to determine the prevalence of gingivitis and its causes in children aged 3 to 7 years.

## 2. Materials and Methods

Approval was obtained from the Bioethics Committee of the Medical University of Warsaw (approval no. KB/190/2016; KB/134/2017; KB135/2019) to conduct the study as part of the monitoring of oral health in children which was conducted in a school setting, in the presence of the parents of the children under study. The study included children attending randomly selected schools located in different provinces of the country. Approximately 8 school or pre-school establishments were selected in each province. The children were surveyed and examined as part of the national oral health monitoring, the 3-year-old age group took place in November 2017, the 5-year-old age group in November 2016, and the 7-year-old age group in November 2019.

Patients in three age groups—3 years old, 5 years old, and 7 years old—were eligible for the study. Inclusion criteria included generally healthy patients, no chronic medication intake, no developmental dental defects, and parents/legal guardians gave and signed informed consent to participate in the study. Patients who were medically compromised, undergoing chronic treatment, taking medication chronically, or presenting signs of developmental defects of dentition, were excluded from the study.

The clinical survey was conducted according to the recommendations and based on the criteria of the WHO classification of clinical conditions (Oral Health Surveys. Basic Methods 5th Edition. WHO Geneva 2013). The questionnaires for parents/legal guardians of children aged 3-, 5- and 7-years contained questions on the following: socio-economic factors (e.g., parents’ education level, economic status, place of residence); questions assessing health behaviors and attitudes regarding oral health (oral hygiene, diet, and limiting the child’s consumption of sugar-containing products, use of dental care, and type of facility; postponement of reporting to the dentist, participation in an educational program on oral health, participation in a supervised brushing program).

The surveys were carried out with the cooperation of regional medical universities and provincial consultants in pediatric dentistry, by teams composed of previously trained and calibrated dentists. The examination was usually conducted by 1 or 3 researchers—dentists who were selected by regional coordinators and agreed to participate. The training and calibration of the investigators took place at the Department of Pediatric Dentistry of the Medical University of Warsaw. The degree of agreement between two measurements was determined using Cohen’s Kappa coefficient. By comparing the results of two consecutive examinations, a determination was made of the magnitude of the error in self-assessment of clinical conditions between the investigators when two or more investigators independently test the same sample, (inter-examiner reliability) and the repeatability of each investigator’s assessment when the investigator tests himself by conducting the survey twice. Evaluation of the investigators’ compatibility with the reference investigator was conducted separately for deciduous and permanent teeth. In examination 1 and 2, Cohen’s Kappa coefficient values for individual investigators ranged from 82.3% to 98.0%. Most investigators achieved very high agreement with the reference investigator (Cohen’s Kappa coefficient as well as the % of concordant assessments were close to 1 or 100%).

The clinical examination assessed the number of teeth present in the oral cavity, the condition of the teeth based on dmft and DMFT indices, and gingivitis based on the presence of bleeding on probing (BOP). Visual and tactile methods using the WHO probe were used for diagnosis. All surfaces of consecutive teeth in both dental arches were evaluated. In children under the age of 12, it is advisable to assess gingival bleeding, as there is a risk of false results around erupting and newly erupted teeth when assessing the depth of gingival pockets around permanent teeth in developmental age. The determination of BOP index was made with WHO probe made of light metal, ending in a 0.5 mm diameter ball. The probe is inserted gently into the gingival pocket, using a force not exceeding 20 g. (A practical test to determine this force is to insert the end of the probe under the nail and apply pressure until fading occurs). When inserting the probe into the pocket and examining the subgingival surface of the tooth, the anatomical shape of the tooth root surface is guided. The occurrence of pain during probe examination indicates the use of too much force.

The obtained results were subjected to statistical analysis (Statistica 13.0 software). Group sizes were based on calculations in Statistica 13. Abundance estimates were calculated assuming: test power = 0.8 and statistical significance level = 0.05. Statistical analysis of the data were carried out to compare the groups under study as well as to assess associations between selected variables. Comparisons of means for quantitative variables between two groups were conducted using the *t*-test (Student’s t) for independent samples. Comparisons of percentages for categorized variables, between two groups, were conducted using the chi-square test of independence. The evaluation of the interaction between the considered parameters was carried out using Spearman’s rank correlation analysis. The significance level was set at *p* < 0.05.

## 3. Results

A total of 3558 patients were examined, of whom 1759 (49.4%) were boys. Table 1 shows the characteristics of the patients, taking into account the age, gender, and dental status (number of teeth, dmft/DMFT indices).

Parents of patients participating in the study most often declared higher education and average economic status. Most of the patients came from urban areas. At least two times a day tooth brushing was declared by 53% of 3-year-olds and more than 65% of 5- and 7-year-olds. In most cases, teeth cleaning was only controlled by the patients’ parents but not performed by them. More than 56% of 3-year-olds had not had a dental visit in the 12 months preceding the survey. Among 5- and 7-year-olds, the most common reason for the last visit was a dental check-up. These data are summarized in Table 2, which also summarizes the frequency of children’s consumption of sugary foods/drinks.

In this study group, gingivitis, diagnosed by the presence of gingival bleeding, was present in 436 patients (120 of 3-year-olds; 156 of 5-year-olds, and 160 of 7-year-olds). Gingivitis was found in 12.25% of the patients participating in the study. In the group of 3-year-olds, gingivitis was significantly more common in boys (*p* = 0.0024). These data are summarized in Table 3.

Spearman’s rank correlation analysis showed significant positive correlations of the presence of gingivitis and the average number of teeth affected by gingivitis with the following: rural residence in the 7-year-old group; male gender in the 5- and 7-year-old groups; dmft/DMFT > 0 or dmft + DMFT or dt/DT values in all age groups; no dental visits in the past 12 months in all age groups; the presence of pain visits in all age groups; and infrequent consumption of fresh fruits and vegetables in the 5- and 7-year-old groups (Table 4).

However, negative significant correlations were found for the following: respondents’ education level in all age groups; brushing at least twice a day and controlled tooth brushing in all age groups; a follow-up visit as the reason for the last dental visit in all age groups; limiting sugar in the diet and eating fresh fruits and vegetables frequently, and designating a day when sweets are eaten in the 5- and 7-year-old groups. The values of the coefficients are summarized in Table 4.

## 4. Discussion

Chronic gingivitis is the most common form of periodontal disease in children and adolescents [10]. Our study showed that gingivitis was present in about an eighth of patients aged 3–7 years. This result is consistent with those obtained by other researchers on a comparable population. Pawlaczyk-Kamienska et al. [3] assessed periodontium in Polish 7-year-olds based on the CPI. Clinically healthy periodontium was noted in 91.32% of patients, while gingival bleeding on probing was noted in 7.46% of patients, compared to 12.25% found in the above study. However, it must be noted that a significant proportion of our patients reported not having had a dental visit in the past year, which may have somehow contributed to the increased frequency of gingivitis in the patients participating in the study. Składnik-Jankowska et al. [11] studied an analogous group of patients, and found the presence of healthy periodontium in 80% of patients, while bleeding gums were found in 14.5% of those evaluated. However, taking into account other circumstances (demographic or socioeconomic differences in the children or adolescents studied), the results of the study seem to vary. Liu et al. [12] found the prevalence of gingivitis in more than 28% of children aged 6–12 years. In studies of social groups with little access to medical care, gingivitis was even observed in more than 60–80% of the subjects [13,14].

In the above study, we did not evaluate the oral hygiene status of the patients based on hygiene indicators, but the parents of the patients determined the method of tooth brushing (self-brushing by the child, controlled or performed by the patient’s parents, and frequency of brushing). Significant negative correlations between brushing teeth at least twice a day and brushing controlled or performed by the parents with the incidence of gingival bleeding in patients were found, from which better oral hygiene in these patients, and thus, less frequent gingivitis can be inferred. A study by Pawlaczyk-Kamienska et al. [3] found a correlation between the amount of bacterial plaque and the likelihood of bleeding, confirming the link between the etiology of gingivitis and the state of oral hygiene. Hygiene in the children participating in this study was rated as sufficient (59.1% of patients) or poor (12.46% of patients). It was determined that children with poor oral hygiene were 25 times more likely to experience bleeding gums than children with good hygiene. Improving daily hygiene routines to remove bacterial plaque appears to be most important in reducing the likelihood of periodontal disease [3].

Some epidemiological studies have shown that gingivitis occurs at a higher frequency in males than in females [6,15]. Some studies suggest that gender may be a risk factor for gingivitis [16]; however, in our study, gingivitis was significantly more common in boys, but only in the 3-year-old group. Differences in the predilection for gingivitis in association with patients’ gender were also not observed in the studies of Liu et al. [12] or Składnik-Jankowska et al. [11].

Among the patients in the above study, significant positive correlations were observed between the presence of dental caries and the number of deciduous or permanent teeth with caries and the incidence of gingival bleeding in all age groups. These observations are confirmed by the work of other researchers. Anusha et al. [17] evaluated the associations of the incidence of dental caries and periodontal disease in childhood patients with Down Syndrome. They found a positive correlation between the DMFT index value and gingivitis (statistically significant) and a positive correlation between the dft component and gingivitis (not statistically significant).

Multivariate regression analysis performed by Folayan et al. [18] found that frequent consumption of refined carbohydrates between meals and plaque index were significantly associated with the presence of moderate to severe gingivitis in adolescents participating in the study. Compared to participants who had a low frequency of carbohydrate consumption between meals, they had more than twice the prevalence of gingival bleeding. In addition, those with higher plaque rates had a 16-fold higher incidence of moderate to severe gingivitis [18]. A relationship between the type of food consumed and the incidence of gingivitis was also observed in our study. Limiting sugar in the diet and frequent consumption of fresh vegetables and fruits negatively correlated with the incidence of gingival bleeding. Above all, brushing teeth at least twice a day has a beneficial effect on the condition of the gums. Poor dietary habits promote the presence of the condition.

To correct the state of the periodontium and prevent gingivitis in children, the combination of home care practices, professional interventions, and behavioral education is essential, such as improving oral hygiene practices, supervising brushing by parents, establishing a healthy diet, and regular dental check-ups. What is more, gingivitis can cause discomfort, swelling, and bleeding, which may affect a child’s ability to eat, speak, and engage in social activities comfortably. Detecting and treating gingivitis early can improve a child’s quality of life by ensuring they remain pain-free. All children and their parents from this study received direct oral health education provided by the researchers during the study. The subjects were provided with information on the benefits of receiving dental care, and ways to prevent oral diseases. Attention was paid to the importance of limiting the consumption of sugary foods, the positive effects on the teeth of cleaning teeth and using fluoride toothpaste, eating fruits and vegetables, and quenching thirst with pure water.

Planning dental care and oral disease prevention programs requires not only diagnosing health problems but also the factors that determine their occurrence or influence their severity, especially sociodemographic determinants and analyzing their changes over time. Thus, it is necessary to conduct regular evaluation of oral health and its determinants by systematic epidemiological studies of selected age groups.

Epidemiological studies are essential for understanding the distribution and determinants of health problems in populations. However, like all scientific approaches, they have their limitations. Recognizing these limitations is crucial for interpreting study results accurately because they are subject to a high risk of bias. In the presented research, relying on questionnaires, data may be inaccurate due to participants’ misreporting or misunderstanding questions and the factor may be considered subjective, even if we tried to measure the reliability of the data. We have tried to avoid errors in the way we collect, record or measure data. We ensured standardized survey conditions, conducting surveys by persons adequately prepared for this (training and calibration) in accordance with the methodology of the World Health Organization but in such extensive research there is always a risk of bias. Finally, the results from our study which was conducted in specific geographic regions, age groups, and socioeconomic backgrounds may not apply to broader populations.

## 5. Conclusions

The prevalence of gingivitis in children aged 3, 5, and 7 years is influenced by socioeconomic, oral hygiene, and dietary factors. Gingivitis may be associated with the male gender. Worse dental status (higher dmft/DMFT ratios) predisposes to gingivitis. Gingivitis is promoted by frequent drinking of sweetened carbonated beverages.

## Figures and Tables

**Table 1:** Characteristics of patients participating in the study.

Age Group	Sex (n/%)		Total	Average Number of Primary Teeth	Average Number of Permanent Teeth	dmft/ DMFT > 0	dmft (Mean)	DMFT (Mean)
	F	M						
3 y.o.	859/52.4%	779/47.6%	1638	19.91 ± 0.53	-------	674 (41.1%)	1.85 ± 3.14	--------
5 y.o.	433 (48.5%)	460 (51.5%)	893	19.43 ± 1.19	0.81 ± 1.68	686/76.9%	4.70 ± 4.33	0.03 ± 0.25
7 y.o.	482 (48.20%)	520 (51.80%)	1002	14.65 ± 2.51	9.17 ± 2.92	814 (81.24%)	4.37 ± 3.53	0.43 ± 0.92

**Table 2:** Data obtained from questionnaires by age group.

Parameter		Age Group (N/%)		
		3 y.o.	5 y.o.	7 y.o.
Education level	Primary	24/1.5%	22/2.5%	36/3.59%
	Secondary/vocational	675/41.2%	412/46.1%	404/40.42%
	Higher	939/57.3%	459/51.4%	561/55.99%
Economic status	Unsatisfactory	192/11.7%	135/15.1%	11/1.10%
	Average	970/59.2%	497/55.7%	766/76.45%
	Very good	476/29.1%	261/29.2%	225/22.46%
Area of living	Urban	866/52.9%	559/62.6%	510/50.50%
	Rural	772/47.1%	334/37.4%	492/49.50%
Teeth brushing at least twice a day		868/53.0%	582/65.2%	656/65.47%
Using mouth rinses			147/16.5%	317/31.64%
Who brushes the child’s teeth?	The child does it on his own	155/9.6%	187/20.9%	378/37.72%
	The parent controls brushing	1220/75.8%	498/55.8%	313/31.24%
	The parent brushes the child’s teeth	566/38.9%	229/32.4%	173/27.72%
Reason for the last dental visit	Has not been to the dentist in the last 12 months or more	926/56.5%	317/35.5%	162/16.2%
	Control visit	605/36.9%	380/42.6%	427/50.12%
	Pain visit	19/6.6%	100/11.2%	132/15.49%
The child eats fresh fruits and vegetables several times a day		276/16.8%	145/16.2%	174/17.37%
Vegetables, fruits served ≤ once a week		89/5.4%	48/4.8%	228/22.7%
Child consumes fruit juices several times a day		175/10.7%	80/8.9%	70/7.8%
Child consumes sugary snacks several times a day		35/2.1%	9/0.9%	33/3.7%
Sweetened carbonated drinks served several times a day		15/0.9%	12/1.3%	23/2.6%
Limiting sugar in the diet		1324/80.8%	721/80.7%	800/79.84%
Designated only one day a week on which the child can consume sweets		138/8.4%	81/11.2%	138/17.25%

**Table 3:** Number and percentages of children with current gingival bleeding and average numbers of teeth with gingival bleeding.

Age Group	Examined Group	Children with Gingival Bleeding N (%)	Teeth with Gingival Bleeding Mean ± SD
3 y.o.	total	120/7.3%	0.35 ± 1.60
	girls	51/5.9%	0.32 ± 1.68
	boys	69/8.9%	0.38 ± 1.51
	*p*	0.024	0.468
5 y.o.	total	156 (17.5%)	1.08 ± 3.14
	girls	74 (17.1%)	1.01 ± 3.06
	boys	82 (17.8%)	1.13 ± 3.22
	*p*	0.772	0.566
7 y.o.	total	160 (15.97%)	0.91 ± 2.93
	girls	78 (16.18%)	1.01 ± 3.28
	boys	82 (15.77%)	0.80 ± 2.57
	*p*	0.8628	0.2563

**Table 4:** Spearman’s rank correlation coefficient (r) between the presence of gingivitis/average number of teeth affected by gingival bleeding and causal factors in the 3-, 5- and 7-year-old age groups.

	3 y.o.		5 y.o.		7 y.o.	
Parameters	Gingival Bleeding (Gingivitis)	Average Number of Teeth with Gingival Bleeding	Gingival Bleeding (Gingivitis)	Average Number of Teeth with Gingival Bleeding	Gingival Bleeding (Gingivitis)	Average Number of Teeth with Gingival Bleeding
Rural area of residence	r = 0.016695; *p* = 0.0831	r = 0.015548; *p* = 0.091	r = 0.0371; *p* = 0.1328	r = 0.0483; *p* = 0.1092	r = 0.062, *p* = 0.0486 *	r = 0.062, *p* = 0.0486 *
Education level	r = −0.050126; *p* = 0.00289 *	r = −0.051093; *p* = 0.0019 *	r = −0.0811; *p* = 0.0018 *	r = −0.0892; *p* = 0.0011 *	r = −0.109, *p* = 0.0006 *	r = −0.111, *p* = 0.0004 *
Unsatisfactory economic status	r = 0.018919; *p* = 0.0771	r = 0.021326, *p* = 0.1002	r = 0.01821; *p* = 0.0811	r = 0.01911; *p* = 0.0981	r = 0.0102; *p* = 0.0891	r = 0.0182; *p* = 0.1312
Male sex	r = 0.012211; *p* = 0.0881	r = 0.043672; *p* = 0.0611	r = 0.09182; *p* = 0.0388 *	r = 0.1039; *p* = 0.0201 *	r = 0.1452; *p* = 0.0021 *	r = 0.1871; *p* = 0.0018 *
Teeth brushing at least twice a day	r = −0.061772; *p* = 0.03652 *	r = −0.07178; *p* = 0.03291 *	r = −0.06721; *p* = 0.0473 *	r = −0.0781; *p* = 0.0391 *	r = −0.062 *p* = 0.0513	r = −0.063 *p* = 0.0459 *
Controlled brushing/brushing performed by parents	r = −0.10111; *p* = 0.0098 *	r = −0.12991; *p* = 0.0029 *	r = −0.0828; *p* = 0.0319 *	r = −0.0782; *p* = 0.0293 *	r = −0.0029; *p* = 0.1293	r = −0.0011; *p* = 0.3921 *
dmft/DMFT > 0	r = 0.136977; *p* = 0.0128 *	r = 0.146794; *p* = 0.0291 *	r = 0.1541; *p* = 0.0003 *	r = 0.1519; *p* = 0.0006 *	r = 0.147 *p* = 0.0000 *	r = 0.146 *p* = 0.0000 *
dmft + DMFT	r = 0.143223; *p* = 0.0229 *	r = 0.140343; *p* = 0.0212 *	r = 0.1872; *p* = 0.0001 *	r = 0.1902; *p* = 0.0001 *	r = 0.251 *p* = 0.0000 *	r = 0.258 *p* = 0.0000 *
dt/DT	r = 0.167860; *p* = 0.021 *	r = 0.168164; *p* = 0.0311 *	r = 0.1291; *p* = 0.0012 *	r = 0.1211; *p* = 0.0026 *	r = 0.1543; *p* = 0.0001 *	r = 0.1557; *p* = 0.0002 *
No dental visit in the last 12 months	r = 0.12911; *p* = 0.0012 *	r = 0.14421; *p* = 0.0019 *	r = 0.14342; *p* = 0.0012 *	r = 0.15473; *p* = 0.0009 *	r = 0.1922; *p* = 0.0001 *	r = 0.2019; *p* = 0.0001 *
Last visit for control	r = −0.09191; *p* = 0.0122 *	r = −0.11911; *p* = 0.01029 *	r = −0.1002; *p* = 0.0091 *	r = −0.1181; *p* = 0.0041 *	r = −0.123 *p* = 0.0003 *	r = −0.126 *p* = 0.0002 *
Last visit because of pain	r = 0.08291; *p* = 0.0201 *	r = 0.070101; *p* = 0.0189 *	r = 0.1038; *p* = 0.0018 *	r = 0.1109; *p* = 0.0039 *	r = 0.1732; *p* = 0.0020 *	r = 0.1717; *p* = 0.0002 *
Fruits and vegetables several times a day	r = −0.00821; *p* = 0.3781	r = −0.001023; *p* = 0.3918	r = −0.1093; *p* = 0.0019 *	r = −0.1129; *p* = 0.0021 *	r = −0.1938; *p* = 0.0001 *	r = −0.1838; *p* = 0.0001 *
Vegetables and fruits served ≤ once a week	r = 0.019391; *p* = 0.30192	r = 0.012991; *p* = 0.3521	r = 0.1119; *p* = 0.0065 *	r = 0.1281; *p* = 0.0027 *	r = 0.2011; *p* = 0.0009 *	r = 0.2011; *p* = 0.0007 *
Limiting sugar in the diet	r = −0.01222; *p* = 0.6467	r = −0.033911; *p* = 0.4929	r = −0.0645; *p* = 0.0271 *	r = −0.0637; *p* = 0.0232 *	r = −0.080 *p* = 0.0116 *	r = −0.089 *p* = 0.0049 *
Designated only one day per week on which the child can consume sweets	r = −0.01010; *p* = 0.3219	r = −0.038281; *p* = 0.2541	r = −0.0637; *p* = 0.0221 *	r = −0.0682; *p* = 0.0291 *	r = −0.1921; *p* = 0.0008 *	r = −0.1847; *p* = 0.0012 *

* statistical significance.

## Data Availability

The data that support the findings of this study are not openly available due to reasons of sensitivity and are available from the corresponding author upon reasonable request. Data are located in controlled access data storage at Medical University of Warsaw.
